# Impact of nanohydroxyapatite on enamel surface roughness and color change after orthodontic debonding

**DOI:** 10.1186/s40510-016-0124-2

**Published:** 2016-04-11

**Authors:** Shabnam Ajami, Hamid Reza Pakshir, Neda Babanouri

**Affiliations:** Orthodontic Research Center, Shiraz University of Medical Science, Shiraz, Iran

**Keywords:** Nanohydroxyapatite, Surface roughness, Debonding

## Abstract

**Background:**

The aim of this prospective in vitro study was to evaluate the effect of nanohydroxyapatite (nanoHAP) serum on the enamel surface roughness and tooth color stability after orthodontic debonding procedure.

**Methods:**

The crowns of 30 premolars were embedded in acrylic blocks with a 4 mm × 5-mm-sized window on the middle third of buccal surfaces. Primary roughness values were evaluated by an atomic force microscope (AFM). After bracket debonding, and polishing procedures, the second roughness parameters were recorded. Specimens were then randomly assigned to two equal groups. NanoHAP serum and HAP toothpaste were applied for 10 days in the first and second groups, respectively. Then, after the third AFM, initial color parameters were measured. Following 1-week immersion in the coffee solution, second color assessment was performed. The fourth AFM was registered after 2 months of aging process.

**Results:**

All roughness parameters were elevated following debonding procedure. There was no statistically significant reduction in roughness parameters after 10 days of nanoHAP serum or HAP toothpaste application. Both groups showed significant color change after immersion in the coffee solution.

**Conclusions:**

NanoHAP serum with the protocols used in this study could not restore enamel surfaces to their original condition.

## Background

The final procedure in the fixed orthodontic treatment is the removal of bonded attachments on the teeth and restoring the enamel surface as closely as possible to the original pretreatment state. Bonding and debonding procedures can cause irreversible changes on the enamel surface [1, 2], which are more important when they occur on the most resistant outer layer. These potential alterations involve up to 55 μm enamel loss [3], increased surface roughness [1, 2], and, therefore, more susceptibility to demineralization and discoloration [4].

Many researchers have evaluated a variety of techniques for bracket debonding, resin removal, and subsequent enamel surface polishing [1, 2, 5–7]. These techniques include using scalers or band-removing pliers [8], different types of tungsten carbide burs in low- or high-speed handpiece followed by water slurry of pumice [9, 10], ultrasonic application and sandblasting techniques [5, 6], fiber-reinforced composite bur [11], Sof-Lex discs [1, 2], and also laser energy to degrade the bonding resin [12]. Nonetheless, there is still no universally accepted method for this potentially conflicting stage of treatment [2].

Moreover, enamel is a non-living tissue which is mainly (97 %) composed of inorganic apatite [13, 14]. In contrast to other hard tissues like bone and dentin, the enamel cannot restore itself [13, 14]. Therefore, synthetic apatites like conventional hydroxyapatite (cHAP) and amorphous calcium phosphate (ACP) have been suggested to repair the damaged enamel due to their close chemical similarities to the enamel structure [15, 16]. These material particles often show different dimensions, morphological characteristics, and orientations from the enamel subunit structures. Therefore, their repairing properties like adsorption to the enamel surface and mechanical strength are compromised [15, 16].

Enamel is made of 20–40-nm-sized particles of hydroxyapatite (HAP) [17, 18]. It has been suggested that 20-nm-sized HAP termed as nanohydroxyapatite (nanoHAP) is the most biocompatible and bioactive form of the synthesized apatites, due to the close similarity to the basic structures of the enamel [15, 16]. NanoHAP has a higher surface area and strong affinity to the enamel surface. These characteristics facilitate its attachment to the enamel surface compared to the large and amorphous types [15, 16]. Interestingly, a layer of nanoHAP formed on the enamel surface is highly resistant to acid solution that subsequently can protect underlying enamel from the future demineralization [15]. Several studies [19–22] have evaluated the effect of nanoHAP on the enamel and dentin remineralization and its preventive potential on dental demineralization. It has been also suggested that the nanoHAP particles could repair bleaching-related microscopic defects of the enamel and thereby improving the post-bleaching sensitivities [23]. Some research teams and manufacturers have proposed that nanoHAP could reduce the enamel surface roughness [24, 25].

As already noted, enamel surface damage is an inevitable sequela of the orthodontic debonding procedure. Moreover, evaluating the potential effects of nanoHAP in restoration of the enamel surface after orthodontic attachment debondings would be crucial for its clinical applications. Hence, the objective of the current in vitro study was to assess the hypothesis that nanoHAP could significantly reduce the enamel surface roughness and the possible discoloration after debonding and polishing procedures.

## Methods

### Sample preparation

Thirty human premolars extracted for orthodontic purposes were selected in 1 month prior to the study. A consent form was signed by each patient, in which it was thoroughly depicted that the enamel surfaces of the teeth extracted for orthodontic treatment would be used in our investigation.

The selection criteria were the absence of cracks, hypoplastic or carious lesions, and restorations on the buccal surfaces of the teeth. The samples were cleaned and stored in distilled water at room temperature. The distilled water was changed weekly to prevent bacterial colonization [2]. Roots of the teeth were removed at the cementoenamel junction by a diamond bur operated in high-speed underwater and air cooling. A rectangular piece of black adhesive tapes (4 mm × 5 mm), corresponding to the bracket bases, was adhered on the middle third of the buccal surface. The crowns were embedded horizontally in the self-cure acrylic resin blocks (Duralay; Reliance Dental C., Worth, IL, USA) in a way that the tape areas were exposed. After the tapes were removed, the exposed enamel windows were cleaned and polished with a low-speed rubber cup and slurry of non-flouridated pumice, washed for 30 s, and dried for 10 s with oil-free air spray.

### Surface roughness assessment

**Fig. 1:**
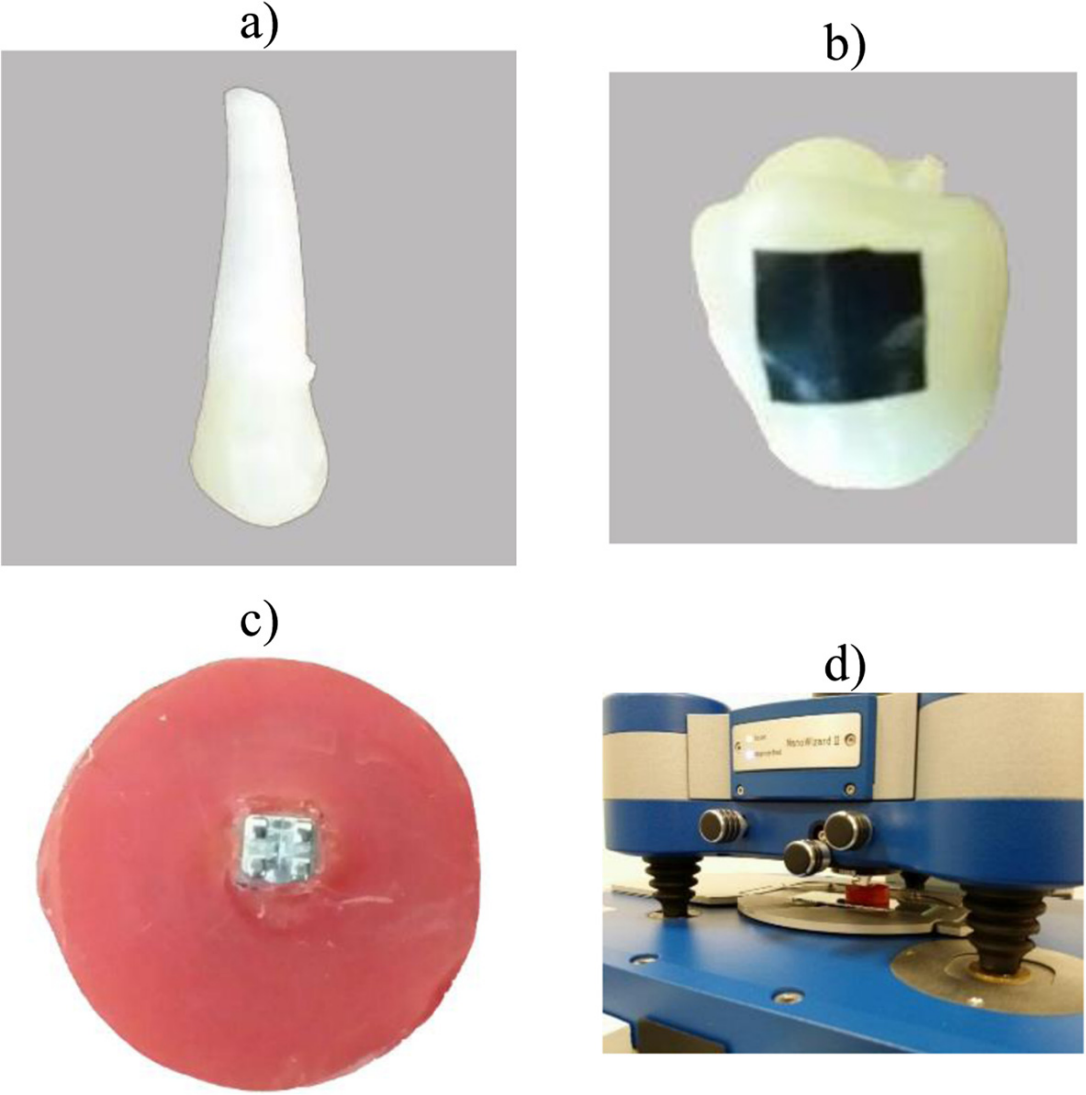
Sample preparation and AFM analysis. A sample prior to preparation (**a**). A rectangular tape to protect the buccal surface before bracket bonding (**b**). A sample with a bonded bracket prior to debonding procedure (**c**). AFM analysis of the sample (**d**)

**Fig. 2:**
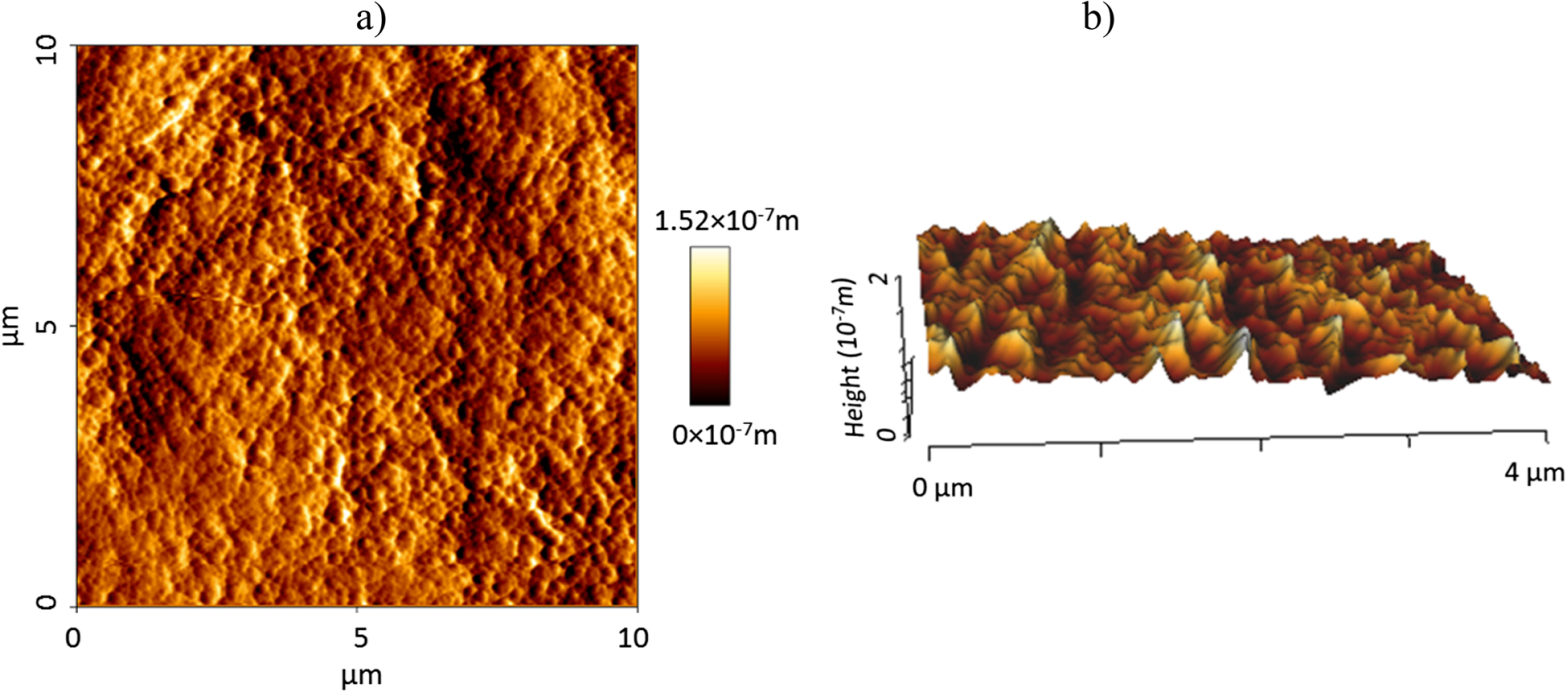
**a** 2D and **b** 3D AFM images (T0)

After coding the samples, they were subjected to the atomic force microscope (AFM) analysis (NanoWizard® II; JPK Instrument AG, Berlin, Germany) to assess initial surface roughness (T0) (Fig. 1). The AFM analysis was performed in the contact mode to make the topographic image from the surface (Fig. 2). The instrument was coupled with a scanner having a maximum range of 100 μm × 100 μm × 15 μm in *x*, *y*, and *z* directions, respectively. Images were acquired at a resolution of 512 × 512 pixels, line rate of 1 Hz, and 10 μm scan size. Three different points were analyzed on the center of each enamel window, and the mean value of these three recoding were used for statistical analysis. A silicone cantilever (HQ: CSC17 MikroMasch®Erope; NanoAndMore GmbH, Wetzlar, Germany) at resonance frequency of 13 kHz with a force constant of 0.18 N/m was used to conduct topographic analysis of the enamel surfaces. Three roughness parameters were registered in nanometers as follows:

- Average roughness value (*R*_a_): the arithmetic mean of the height of peaks and depth of the valleys from a mean line. This parameter describes the overall surface roughness.
- Root mean square roughness (*R*_q_): the height distribution relative to the mean line.
- *R*_z_: the average maximum peak-to-valley height of five successive sample lengths.

### Bonding, debonding, and resin removal

After initial evaluation, all teeth were subjected to a 37 % phosphoric acid gel (Etching agent; Reliance, Itasca, IL, USA) for 30 s, thoroughly rinsed and dried with oil-free air spray for 15 s. A thin layer of primer (Transbond XT; 3M Unitek, Monrovia, CA, USA) was applied on the etched surfaces, and adhesive resin was placed on a stainless steel standard edgewise premolar bracket bases (Dentaurum; Ispingen, Germany). The brackets were placed on the enamel windows, firmly pressed in place, and excessive resin was removed and light cured for 10 s from each edge of the brackets, for a total exposure time of 40 s (Fig. 1).

The samples were stored in distilled water at room temperature for 24 h to ensure complete resin polymerization. Brackets were peeled off with a hand plier by gently squeezing the mesial and distal wings together. Remnant resin was removed with 12-fluted tungsten carbide bur (Dentaurum no. 123-604; Ispingen, Germany), operated in low-speed and air cooling, followed by a PoGo polisher (Dentsply Caulk, Milford, DE, USA), and finally polished with a rubber cup and slurry of fine pumice for 10 s. Complete removal of residual resin was confirmed by visual inspection under the light of a dental operating lamp and ×10 magnification. All of the above procedures on all samples were performed by an orthodontist, and a new bur, polisher, and rubber cup were used for each sample. After completion of the cleanup procedures, the second AFM was taken to register roughness parameters (T1). Then, the teeth were randomly assigned to two equal groups (*n* = 15) using the table of random numbers. In the first group, a high concentration nanoHAP serum (n-HAP Repairing Serum; PrevDent International BV, Netherlands) was applied on the enamel surface for 2–3 min by the sponge at the head of the tubes (Fig. 1) and rinsed after 20 min with water. The process was performed once a day for 10 days according to the manufacturer’s instructions. Specimens in the HAP toothpaste group were manually brushed with horizontal technique for 20 s, using a new soft brush for each sample and HAP-contained toothpaste (Signal Expert Protection; Unilever France, Rueil-Malmaison Cedex, France), daily during these 10 days. Thereafter, the third AFM evaluation of roughness parameters (T2) was obtained blindly by a second investigator.

### Color stability assessment

Using a spectrophotometer (VITA Easyshade Advance 4.0; VITA Zahnfabrik H, Rauter Gmbh & Co., Bad Säckingen, Germany), both study groups were subjected to the spectrophotometric assessment to measure the primary color of the enamel surface. Color quantification was according to the CIE lab system (Commision Internatinale de l’Eclairage, L*, a*, b*) presented in Table 1. Before color assessment, all samples were cleaned with the soft brush and water for 10 s and dried with absorbent paper. The spectrophotometer was calibrated before each imaging with the white pad supplied by the manufacturer. The spectrophotometric evaluation of each sample was recorded two consecutive times. When the total color difference (Δ*E*) of two measurements did not exceed the threshold of 1 Δ*E* unit, the mean value of these measurements was used for statistical analysis. Measurements with Δ*E* > 1 were discarded, and new evaluation were performed a second time. The color measurements were repeated after 1-week immersion of all samples in the coffee solution at room temperature. The coffee solution was prepared by 15 g coffee (Nescafé Blend 43; Nestlé Australia ltd, NSW, Australia) in 500 mL boiling water and changed everyday [26]. All measurements were performed in the same room blindly by one investigator, while the samples were placed over a white paper to provide white background. The CIE values (L*, a*, b*) were obtained, and the data were analyzed regarding their lightness and chromaticity values (Δ*L*, Δ*a*, Δ*b*). The total color change (Δ*E*) between two intervals was calculated according to the following equation:

$$ \varDelta E = {\left[{\left(\varDelta L\right)}^2 + {\left(\varDelta a\right)}^2+{\left(\varDelta b\right)}^2\right]}^{1/2} $$

**Table 1:** CIE color system

Color parameter	Definition	Color range
L*	Measure of lightness (value)	Ranging from black (0) to white (100)
a*	The position on red-green axis	Ranging from red (+) to green (−)
b*	The position on yellow-blue axis	Ranging from yellow (+) to blue (−)

After color evaluation, both groups were stored in the distilled water, weekly changed, at the room temperature for additional 2 months, and finally subjected to AFM analysis (T3) to assess long-term effect of nanoHAP.

### Statistical analysis

Statistical analysis was performed with the Statistical Package for Social Sciences (Version 15.0, SPSS Inc., Chicago, IL, USA). The assumption of normality was investigated by Kolmogorov-Smirnov test. Data for roughness parameters were statistically evaluated with repeated measurements analysis of variance (RM-ANOVA). Changes in color parameters (L*, a*, b*) in each group were investigated by paired sample *t* test. Student’s *t* test was further used for evaluating roughness, differences of the total color change (Δ*E*), and also (ΔL*, Δa*, Δb*) among the groups. Significance was set at probability value of *P* < 0.05 for all tests.

## Results

**Fig. 3:**
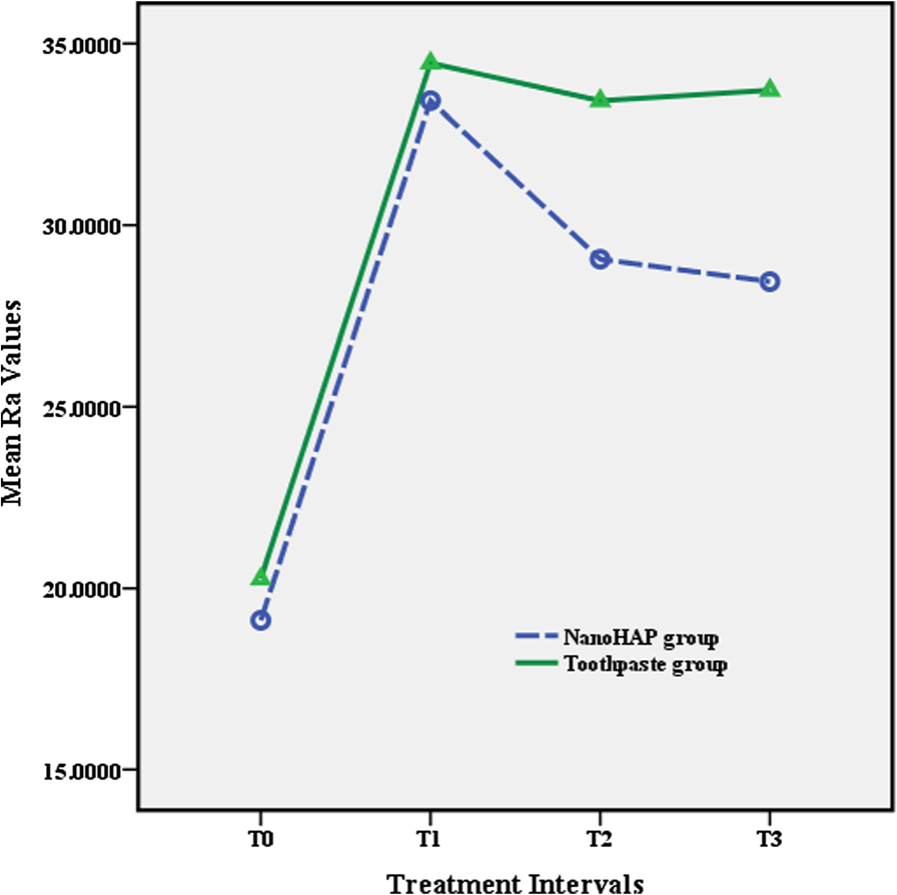
Results of average roughness (*R*_a_)

**Fig. 4:**
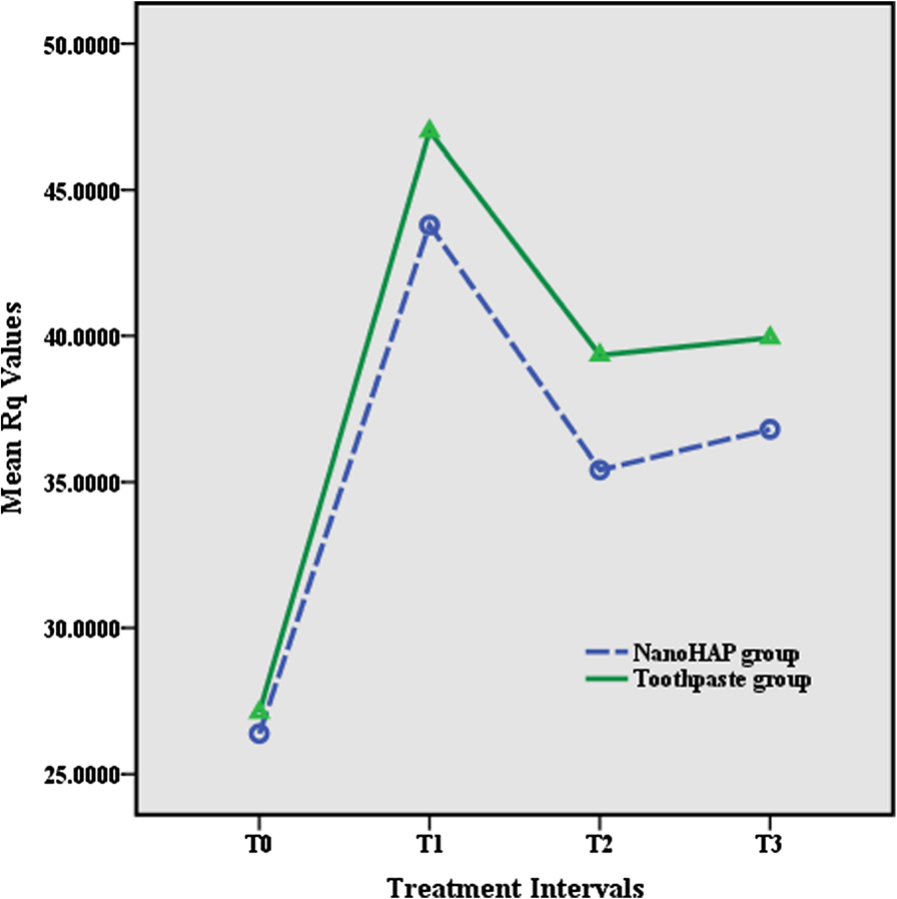
Results of root mean square roughness (*R*_q_)

**Fig. 5:**
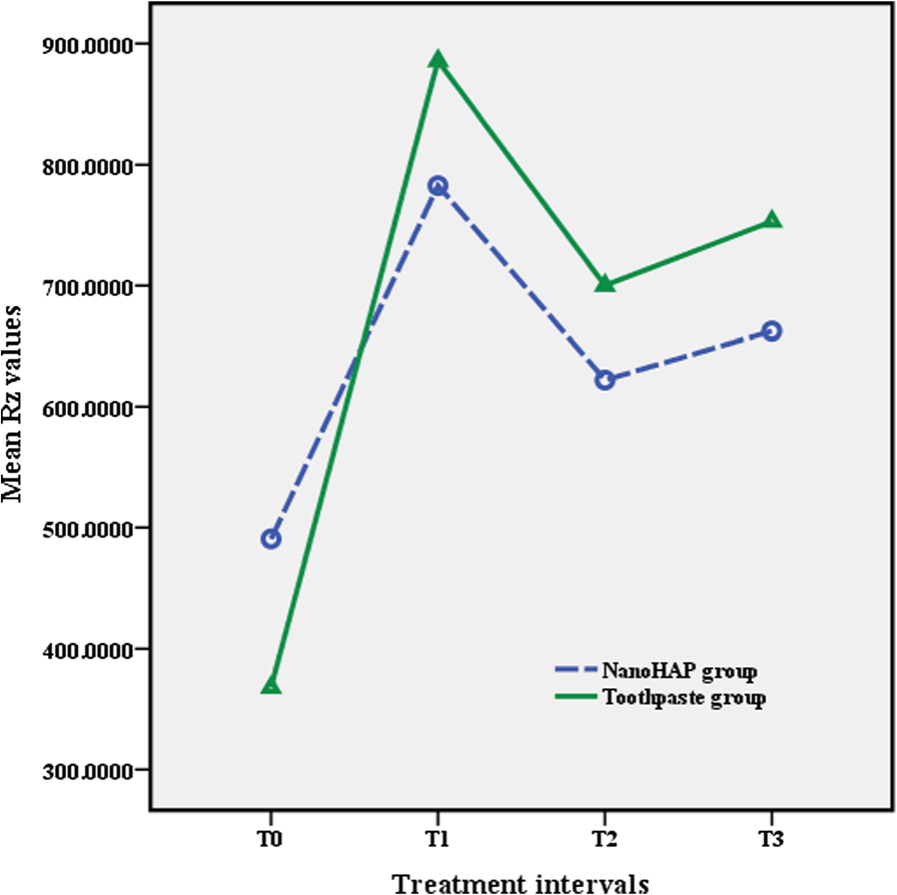
Results of maximum peak-to-valley height (*R*_z_)

The RM-ANOVA showed that there was no significant interaction effect between treatment and time variables for all roughness parameters (all *P* > 0.05). Figures 3, 4, and 5 depict the mean profile changes in *R*_a_, *R*_q_, and *R*_z_ throughout the treatment intervals, respectively.

The results of the *t* test indicated that there was no significant difference between the two study groups in all treatment intervals. The results revealed irreversible enamel surface changes in both study groups following debonding procedures. All roughness parameters increased significantly from T0 to T1 in both experimental groups (both *P* < 0.001), whereas there were no statistically significant differences between T1, T2, and T3 (Table 2).

**Table 2:** Means, standard deviation, and *t* test results of surface roughness measurements (nm) for the groups

Roughness parameter		T0	T1	T2	T3
*R*_a_	NanoHAP	19.1 ± 5.4^A^	33.4 ± 13.6^B^	29.9 ± 12.4^B^	28.4 ± 9.9^B^
	Toothpaste	20.2 ± 7.1^A^	34.4 ± 12.4^B^	33.4 ± 13.2^B^	33.7 ± 12.4^B^
	*P* value*	0.62	0.83	0.36	0.21
*R*_q_	NanoHAP	26.3 ± 8.1^A^	43.7 ± 15.9^B^	35.4 ± 13.3^B^	36.7 ± 12.1^B^
	Toothpaste	27.1 ± 8.7^A^	46.9 ± 17.7^B^	39.3 ± 14.4^B^	39.9 ± 14.5^B^
	*P* value*	0.81	0.6	0.44	0.52
*R*_z_	NanoHAP	490.5 ± 188.2^A^	782.7 ± 385.8^B^	622.0 ± 271.9^B^	622.4 ± 260.9^B^
	Toothpaste	368.0 ± 195.3^A^	855.6 ± 434.3^B^	700.1 ± 426.1^B^	753.1 ± 377.1^B^
	*P* value*	0.09	0.49	0.55	0.45

In each row, mean values with the same capital letter in superscript were not statistically different (Sidak test)

*T0* prebond, *T1* after resin removal, *T2* after 10 days of nanoHAP serum or HAP toothpaste application, *T3* after 2 months

*t test results

The results of color assessment showed that L* parameter significantly decreased after immersion in the coffee solution in both nanoHAP group and HAP toothpaste group (*P* = 0.004 and 0.001, respectively). There was no significant change in a* whereas b* increased statistically significant (*P* < 0.001) in both groups (Table 3).The results indicated that there were significant differences in the average values of Δa* and Δb* between groups. No significant difference in terms of total color change (Δ*E*) was indicated between study groups, although this difference was very close to the adopted significant level (*P* = 0.06). The clinical significance of the color changes was determined by comparing the differences in color values with the proposed standard value of clinical detection which normally is set to 3.7 Δ*E* units [27]. It was found that 80.0 % of the teeth in the HAP toothpaste group had shown visible and clinically significant color changes while only 46.7 % of teeth in the nanoHAP group had demonstrated visible changes. When considering the number of teeth with visible color change, difference among study groups tended to be significant (*P* = 0.058).

**Table 3:** Means and standard deviation of ΔL*, Δa*, Δb*, Δ*E*, and *t* test results of the groups

	ΔL*	Δa*	Δb*	Δ*E*
NanoHAP	0.93 ± 1.03	0.84 ± 1.71	3.17 ± 1.69	3.94 ± 1.63
Toothpaste	1.40 ± 1.35	0.30 ± 0.94	4.39 ± 1.11	4.91 ± 0.99
*P* value*	0.29	0.03	0.02	0.06

*t test results

## Discussion

The substantial clinical significance of outer layer of enamel brings imperative concern over orthodontic bonding and debonding procedures. The hard layer with high content of minerals and fluoride protects the underlying enamel from organic acids produced in dental microbial plaque [28]. In addition, increased enamel surface roughness after debonding results in increased accumulation of the pigments, retention of bacterial plaque, and decalcification, both situation that may cause esthetic problems [29, 30]. Previous studies reported inevitable enamel alterations including enamel loss [3, 31], increased surface roughness [1, 2], and discoloration [32, 33], following orthodontic treatments.

Benefiting from various methods or biomaterials that help restore enamel surface to the original condition is crucial. Receiving more interest in dental practice, nanoHAP with its chemical and structural similarity to the enamel inorganic structure, has shown to be superior to the conventional synthetic HAP in repairing damaged enamel [15]. The nanoscale HAP exhibits high surface energy and strong affinity to the enamel surface [15, 16]. In the present study, the effects of nanoHAP on the enamel surface roughness and color stability after orthodontic debonding were investigated and compared with the HAP-contained toothpaste.

The results of the current study showed that all the roughness variables elevated significantly following the debonding and polishing procedures which was in accordance to the results yielded by previous studies [1, 2]. The results also revealed that 10-day application of the nanoHAP serum did not significantly reduce the enamel surface roughness. There was no difference between the study groups regarding the surface roughness in all treatment intervals. These results were in contrast to the findings of Takikawa et al. [25] and also Toko et al. [24] who reported that nanoHAP reduced bleaching-related roughness and restored the enamel surface to the original state, though they evaluated the effect of nanoHAP on the chemically induced roughness. One possible explanation is that in these studies, roughness was induced by chemical procedure, whereas in our study, nanoHAP was used for mechanically induced roughness.

In the current study, completion of resin removal was verified with visual inspection under dental operating light and ×10 magnification; nonetheless, remnant of small particles of adhesive would be inevitable. Although randomization after resin removal made this factor equal in both experimental groups, the presence of remnant resin may interfere with adsorption of nanoHAP particles. Moreover, these remnants might have increased the roughness parameters and masked the potential effect of nanoHAP serum.

In the present study, AFM was employed to evaluate surface topographic characteristics of the enamel which was considered as an accurate technique for providing 3D detailed topographic definition of the surface roughness at the nanometer level [34]. Providing the quantitative information of the roughness is the main advantage of AFM over other methods like scanning electron microscopy (SEM) [35]. Moreover, AFM needs minimal sample preparation and allows for reexamination of the sample [11, 34]. However, one of its shortcomings is related to the very small size of the analyzed areas representing the large prepared surfaces in all samples [36]. To minimize this limiting factor in the current study, at least three different points were analyzed for each sample and the mean value of these measurements were used for the statistical analysis.

Surface topography is inherently three dimensional; therefore, studies which have employed two-dimensional technologies or used only *R*_a_ value as a roughness indicator may have not registered the precise characteristics of the surface [34, 37]. Surfaces with significantly different roughness features may possess identical *R*_a_ since this parameter cannot indicate the depth of the irregularities or differentiate between pores and projections [37, 38]. Additional roughness variables are needed to enhance the description of the surface profile. In the present study, *R*_a_, *R*_q_, and *R*_z_ were used to indicate enamel surface roughness; the changes of these variables were similar in both groups at different time intervals.

Another concern over orthodontic bonding and debonding procedures is related to their impacts on tooth color alteration and increased susceptibility to exogenous pigmentations and thus esthetic impairment of the enamel surface [26, 32, 33]. Various etiologic factors are responsible for the tooth color alteration after debonding such as endogenous and exogenous discoloration of penetrated resin tags [33, 39]. The debonding-induced surface roughness should be addressed as one of the other factors responsible for the enamel color alteration. Alteration in light reflection and optical properties of the enamel surface [32] together with increased retention of exogenous pigments [29] are considered to be the two related factors in enamel color changes after debonding procedures.

Based on the assumption that nanoHAP reduces enamel surface roughness, its potential positive effect on the color stability following orthodontic debonding was investigated in the present study. Our results showed that 10-day application of nanoHAP serum did not significantly reduce the amount of enamel color discolorations compared to the HAP toothpaste group. This finding was in accordance with the outcome surface roughness analysis. In addition, the absorption of coffee pigments by remnant resin tags may outweigh any probable differences in enamel surface characteristics. Both groups in this investigation indicated significant change in L*, a*, and b* following 1-week immersion in coffee solution. Generally, the mean L* value decreased while the mean b* increased in both groups representing darker and more yellowish color for the teeth. The mean values of total color change (Δ*E*) were greater than 3.7 units, a standard value of clinical detection [27], in both groups. However, differences between the groups with respect to the mean Δ*E* value and also the number of teeth which exceeded this threshold value (Δ*E* > 3.7) tended to be significantly lower in the nanoHAP group. With a larger sample size, the color parameters may reach the significant level.

In the present study, we found that nanoHAP serum had no significant effect on debonding-induced enamel surface roughness and tooth color stability. It could be hypothesized that for the mechanically induced roughness, nanoHAP with higher concentrations or endured application would be more effective. This opinion can be examined in a future study. It may also be interesting to investigate the effect of nanoHAP on the mechanically induced roughness without the adhesive resin as an interfering factor.

## Conclusions

It could be concluded that orthodontic bonding and debonding procedures increased enamel surface roughness. The nanoHAP serum and HAP toothpaste tested in the present study could not reduce enamel surface roughness parameters to restore enamel to its original roughness condition.

Although there were fewer teeth with clinically visible color change in the nanoHAP group, 10-day application of nanoHAP could not improve enamel color stability after orthodontic debonding.
